# Serum free fatty acids are associated with severe coronary artery calcification, especially in diabetes: a retrospective study

**DOI:** 10.1186/s12872-021-02152-w

**Published:** 2021-07-15

**Authors:** Yangxun Xin, Junfeng Zhang, Yuqi Fan, Changqian Wang

**Affiliations:** Department of Cardiology, Shanghai Ninth People’s Hospital, Shanghai Jiao Tong University School of Medicine, Shanghai, China

**Keywords:** Free fatty acids, Coronary arteries, Calcification, Intravascular ultrasound, Diabetes mellitus

## Abstract

**Background:**

Serum free fatty acid (FFA) concentrations are associated with coronary heart disease and diabetes mellitus (DM). Few studies focused on the relationship between serum FFA levels and coronary artery calcification (CAC).

**Methods:**

This was a retrospective, single-centered study recruiting patients underwent FFA quantification, coronary angiography and intravascular ultrasound (IVUS). CAC severity was assessed with the maximum calcific angle (arc) of the calcified plaque scanned by IVUS. Patients with an arc ≥ 180° were classified into the severe CAC (SCAC) group, and those with an arc < 180° were classified into the non-SCAC group. Clinical characteristics, serum indices were compared between 2 groups. Logistic regression, receiver operating characteristic (ROC) curves and area under the curves (AUC) were performed.

**Results:**

Totally, 426 patients with coronary artery disease were consecutively included. Serum FFA levels were significantly higher in the SCAC group than non-SCAC group (6.62 ± 2.17 vs. 5.13 ± 1.73 mmol/dl, *p* < 0.001). Logistic regression revealed that serum FFAs were independently associated with SCAC after adjusting for confounding factors in the whole cohort (OR 1.414, CI 1.237–1.617, *p* < 0.001), the non-DM group (OR 1.273, CI 1.087–1.492, *p* = 0.003) and the DM group (OR 1.939, CI 1.388–2.710, *p* < 0.001). ROC analysis revealed a serum FFA AUC of 0.695 (CI 0.641–0.750, *p* < 0.001) in the whole population. The diagnostic predictability was augmented (AUC = 0.775, CI 0.690–0.859, *p* < 0.001) in the DM group and decreased (AUC = 0.649, CI 0.580–0.718, *p* < 0.001) in the non-DM group.

**Conclusions:**

Serum FFA levels were independently associated with SCAC, and could have some predictive capacity for SCAC. The association was strongest in the DM group.

**Supplementary Information:**

The online version contains supplementary material available at 10.1186/s12872-021-02152-w.

## Background

Coronary artery calcification (CAC) has been traditionally recognized as a common complication in aging patients, and those with diabetes mellitus (DM) or chronic kidney disease (CKD) [1, 2]. CAC was observed in over 90% of men and 67% of women older than 70 years [3, 4]. The extent of CAC strongly correlates with the degree of atherosclerosis [5], and can predict future cardiovascular events [6–8]. Severe coronary artery calcification (SCAC), which indicates an extensively progressed calcified plaque typically with a calcified angle > 180° surrounding the endothelium of the coronaries, remains a challenge for percutaneous coronary intervention (PCI). Clinical experience showed that SCAC poses a risk of failure in device delivery, coronary dissection, or insufficient expansion of the stent. Common methods to assess CAC include computed tomography (CT), coronary angiography (CAG), intravascular ultrasound (IVUS) and optical coherence tomography (OCT) [9]. No effective pharmacotherapies have been confirmed to reverse the process of CAC.

Serum free fatty acids (FFAs) are one of the sources of energy in the body. They were found to be associated with insulin resistance and the development of DM [10–13]. Previous work elucidated that serum FFAs were an independent risk factor for cardiovascular events both in stable coronary artery disease (CAD) [14, 15] and in acute coronary syndrome [16, 17]. Serum FFA levels were also associated with prognosis in acute heart failure [18] and the incidence of heart failure in aged patients [19]. The underlying mechanism of the adverse cardiovascular prognosis of FFAs remains unknown. Moreover, whether FFAs exert a negative influence on coronaries by promoting the process of CAC needs to be investigated. Numerous studies have shown that FFAs (especially saturated FFAs) induce vascular calcification [20–25]. Few clinical studies focused on the relationship between serum FFA levels and arterial calcification [10, 22, 26], but with inconsistent conclusions.

Therefore, we attempted to explore the potential relationship between serum FFA levels and CAC in the present study.

## Methods

### Study design and participants

This was a single-center, retrospective study conducted at the Shanghai Ninth People's Hospital affiliated with Shanghai Jiao Tong University School of Medicine from February 2017 to February 2020. The enrollment criteria were as follows: (1) patients aged ≥ 18 years, and (2) patients who underwent CAG and IVUS for a de novo lesion and were diagnosed with CAD. The exclusion criteria were as follows: (1) previous PCI or coronary artery bypass grafting (CABG) on the target vessel; (2) patients with a lesion of chronic total occlusion; (3) moderate or severe cardiac valve disease; (4) NYHA class 3–4 heart failure; (5) type 1 DM (T1DM); (6) patients with a declined renal function of eGFR ≤ 30 ml/min/1.73 m^2^ or who underwent hemodialysis; (7) severe hepatic dysfunction; (8) malignant tumor; or (9) patients with poor quality IVUS imaging or lack of laboratory test results. All patients gave written informed consent, and the study protocol was approved by the Institution's Human Investigation Committee. Procedures were performed in accordance with the Declaration of Helsinki.

### Demographic data and blood test

Detailed medical history of the patients was collected after hospitalization. Weight and height were recorded and body mass index (BMI) was calculated by weight/height^2^ (kg/m^2^). Blood pressure was measured on admission. Blood samples were routinely collected in the morning after overnight fasting when hospitalized, including lipid profiles (serum FFA was part of the fasting lipid profiles), kidney function, HbA1c et al. Estimated glomerular filtration rate (eGFR) were calculated according to CKD-EPI equation.

### CAG and IVUS procedures

The CAG procedures were performed according to generally accepted guidelines and routines [27, 28]. The radial artery was the preferred access approach. Lesions were imaged in at least two different projections, preferably at 90°. A lesion with a reduced luminal diameter of at least 50% was considered significant. All procedures were performed by two experienced interventional cardiologists, with sub-senior title or higher. Both the cardiologists decided together whether or not an IVUS procedure was needed after CAG. After intracoronary administration of 200 μg of nitroglycerin, pre-intervention IVUS imaging of all the coronaries was performed using a commercially available IVUS system and catheter (iLab™ Ultrasound Imaging System, Boston Scientific Corp. Natic, MA, USA; Opticross™ 3.0 F intracoronary ultrasound catheter, Boston Scientific). The IVUS catheter was placed at least 10 mm distal to the lesion and then moved backward automatically at a speed of 0.5 mm/s until it reached the coronary ostium.

### IVUS analysis

All IVUS data were stored in DVDs and analyzed offline according to the criteria of the American College of Cardiology Clinical Expert Consensus Document on Standards for Acquisition, Measurement and Reporting of Intravascular Studies [29]. Analyses were independently performed with echo plaque software (Indec Medical Systems, Santa Clara, CA) by two experienced interventional cardiologists who were blinded to all the patients’ characteristics. From all the coronaries, we selected the vessel with the most severe lesion and the most severe calcified plaque as our targets. In the case of multiple lesions within a single coronary segment, distinct lesions or stenosis had at least 5 mm distance between them, with the most severe lesion as our target. The lesion length was measured. The reference sites were the most normal-appearing positions within 5 mm proximal and distal to the lesion border but before any side branch, and were used to calculate a mean reference cross-sectional area (CSA). The minimum lumen CSA sites were the slices with the smallest lumen CSA. Quantitative analysis included the measurement of the external elastic membrane (EEM) and lumen area (LA) in the minimum lumen area (MLA) position. Plaque plus media CSA was calculated as MLA-EEM minus MLA-LA. Plaque burden was calculated as plaque and media CSA divided by MLA-EEM multiplied by 100%.

Coronary calcium was defined as a brighter plaque than adventitia with acoustic shadowing. The arc of the calcified plaque in the lesion was measured. Calcium length was determined as the length of the calcified plaque with an arc. According to the arc value, the cohort was divided into two groups: (1) Patients with an arc ≥ 180° were classified into the SCAC group, and those with a calcified arc < 180° or no calcium were classified into the non-SCAC group.

### Statistical analysis

Statistical analysis was performed with SPSS version 20.0 (IBM Corp., Armonk, NY, USA). Continuous variables are expressed as the Mean ± SD for normally distributed variables. Categorical data are presented as frequencies and proportions. Continuous variables were assessed using unpaired Student's t-tests, while categorical variables were compared using chi-square tests. Pearson’s correlation analyses between serum FFAs, and clinical and IVUS data were performed. Binary logistic regressions were used to calculate the correlations between the confounders and SCAC. Parameters were considered potential confounders if associations were found with a *p-*value < 0.10 in single factor analysis. Receiver operating characteristic (ROC) curves were constructed to assess the diagnostic value of FFAs according to the area under the curve (AUC). All *p-*values and confidence intervals (CI) were two-sided, and *p* < 0.05 was considered statistically significant.

## Results

### Baseline clinical characteristics

Totally, 495 patients who underwent both CAG and IVUS were analyzed. A total of 69 patients were excluded, including 36 cases for previous PCI on target vessel, 2 for CABG of the target, 6 for severe heart failure, 1 for T1DM, 5 for declined renal function, 5 for cancer, 9 for insufficient lab results and 5 for poor quality IVUS. Finally, 426 vessels from 426 patients (SCAC group: 106, 24.9%) were included. Table 1 shows that patients in the SCAC group were older (71.74 ± 9.44 vs. 65.62 ± 10.11 years, *p* < 0.001), more likely to have a history of DM (37.7% vs. 26.2%, *p* = 0.024), elevated HbA1c (6.59 ± 1.48 vs. 6.26 ± 1.33, *p* = 0.031), increased pulse pressure (62.41 ± 15.07 vs. 54.55 ± 12.55 mmHg, *p* < 0.001) and decreased renal function (76.00 ± 20.56 vs. 82.51 ± 20.64 ml/min/1.73 m^2^, *p* = 0.005). Serum FFA levels were significantly increased in the SCAC group (6.62 ± 2.17 vs. 5.13 ± 1.73 mmol/dl, *p* < 0.001). No significant differences in clinical presentation, serum cholesterol levels, total triglyceride levels, or use of drugs were observed between the two groups (Table 1).

**Table 1:** Baseline clinical characteristics

Variable	Total (n = 426)	Non-SCAC (n = 320)	SCAC (n = 106)	*P* value
Age, years	67.14 ± 10.29	65.62 ± 10.11	71.74 ± 9.44	< 0.001
Male sex, %	281 (66.0)	213 (66.6)	68 (64.2)	0.650
BMI, kg/m^2^	24.40 ± 2.84	24.44 ± 2.80	24.29 ± 2.98	0.653
Smoking, %	221 (51.9)	170 (53.1)	51 (48.1)	0.371
Family history, %	103 (24.2)	75 (23.4)	28 (26.4)	0.535
Hypertension, %	303 (71.1)	221 (69.1)	82 (77.4)	0.102
Diabetes, %	124 (29.1)	84 (26.2)	40 (37.7)	0.024
Prior MI, %	42 (9.9)	30 (9.4)	12 (11.3)	0.560
Prior PCI %	90 (21.1)	62 (19.4)	28 (26.4)	0.124
*Clinical presentation*				0.980
SCAD	354 (83.1)	266 (83.1)	88 (83.0)	
ACS	72 (16.9)	54 (16.9)	18 (17.0)	
eGFR, ml/min/1.73m^2^	80.89 ± 20.79	82.51 ± 20.64	76.00 ± 20.56	0.005
HbA1c, %	6.34 ± 1.38	6.26 ± 1.33	6.59 ± 1.48	0.031
TG, mmol/L	1.63 ± 0.89	1.66 ± 0.87	1.54 ± 0.94	0.204
TC, mmol/L	4.17 ± 1.01	4.14 ± 0.97	4.24 ± 1.14	0.381
HDL, mmol /L	1.06 ± 0.26	1.05 ± 0.26	1.07 ± 0.28	0.465
LDL, mmol/L	2.66 ± 0.85	2.63 ± 0.82	2.74 ± 0.94	0.230
Serum FFAs, mmol/dl	5.51 ± 1.96	5.13 ± 1.73	6.62 ± 2.17	< 0.001
SBP, mmHg	131.50 ± 17.02	130.03 ± 16.03	135.93 ± 19.10	0.002
DBP, mmHg	75.01 ± 10.52	75.48 ± 10.38	73.62 ± 10.89	0.116
PP, mmHg	56.51 ± 13.63	54.55 ± 12.55	62.41 ± 15.07	< 0.001
Prior medical treatment				
Anti-platelet drugs (n/%)	119 (28.0)	84 (26.3)	35 (33.0)	0.184
Statins (n/%)	99 (23.2)	73 (22.8)	26 (24.5)	0.717
ACEI/ARB (n/%)	194 (45.5)	139 (43.4)	55 (51.9)	0.130
β-blockers (n/%)	81 (19.0)	55 (17.2)	26 (24.5)	0.095
CCB (n/%)	144 (33.8)	102 (31.9)	42 (39.6)	0.144
Insulin (n/%)	32 (7.5)	21 (6.6)	11 (10.4)	0.197

Data are expressed as the mean ± SD. BMI, body mass index; MI, myocardial infarction; PCI, percutaneous coronary intervention; SCAD, stable coronary artery disease; ACS, acute coronary syndrome; eGFR, estimated glomerular filtration rate; TC, total cholesterol; TG, triglyceride; HDL, high-density lipoprotein cholesterol; LDL, low-density lipoprotein cholesterol; SBP, systolic blood pressure; DBP, diastolic blood pressure; PP, pulse pressure; ACEI/ARB, angiotensin-converting enzyme inhibitors/angiotensin receptor blockers; CCB, calcium channel blockers

### CAG and IVUS results

The distribution of SCAC in the coronaries was similar to that in the non-SCAC group (Table 2). There were no significant differences in MLA-EEM and reference EEM between the two groups. Patients with SCAC tended to have a severe lesion with an increased lesion length (33.73 ± 13.25 vs. 18.91 ± 10.0 mm, *p* < 0.001), a smaller MLA-LA (3.82 ± 1.68 vs. 4.92 ± 2.79 mm^2^, *p* < 0.001) and a higher plaque burden (0.73 ± 0.08 vs. 0.67 ± 0.11, *p* < 0.001) than the non-SCAC patients. Calcium length (11.36 ± 5.73 vs. 4.97 ± 3.44 mm, *p* < 0.001) and calcium arc (290.56 ± 62.91 vs. 90.03 ± 41.42°, *p* < 0.001) were also increased in the SCAC group (Table 2).

**Table 2:** Angiographic and IVUS analysis of lesion characteristics

Variable	Total (n = 426)	Non-SCAC **(**n = 320**)**	SCAC (n = 106)	*P* value
*Target vessel*				0.066
LM	14 (3.3)	9 (2.8)	5 (4.7)	
LAD	311 (73.0)	226 (70.6)	85 (80.2)	
LCX	31 (7.3)	28 (8.8)	3 (2.8)	
RCA	70 (16.4)	57 (17.8)	13 (12.3)	
Lesion length (mm)	22.59 ± 12.63	18.91 ± 10.0	33.73 ± 13.25	< 0.001
Reference EEM (mm^2^)	14.39 ± 4.46	14.33 ± 4.58	14.59 ± 4.06	0.597
MLA-CSA (mm^2^)	14.46 ± 4.55	14.45 ± 4.69	14.50 ± 4.12	0.919
MLA-LA (mm^2^)	4.65 ± 2.60	4.92 ± 2.79	3.82 ± 1.68	< 0.001
Plaque area (mm^2^)	9.83 ± 3.52	9.57 ± 3.53	10.59 ± 3.40	0.010
Plaque burden	0.69 ± 0.11	0.67 ± 0.11	0.73 ± 0.08	< 0.001
Calcium length (mm)	7.18 ± 5.32	4.97 ± 3.44	11.36 ± 5.73	< 0.001
Calcium arc (°)	159.49 ± 107.78	90.03 ± 41.42	290.56 ± 62.91	< 0.001

Data are expressed as the mean ± SD. LM, left main coronary; LAD, left anterior descending branch; LCX, left circumflex branch; RCA, right coronary artery; EEM, external elastic membrane; MLA-CSA, cross-sectional area in the minimal lumen area site; MLA-LA, luminal area in the minimal lumen area site

### Correlation between serum FFAs and clinical and IVUS data

**Fig. 1:**
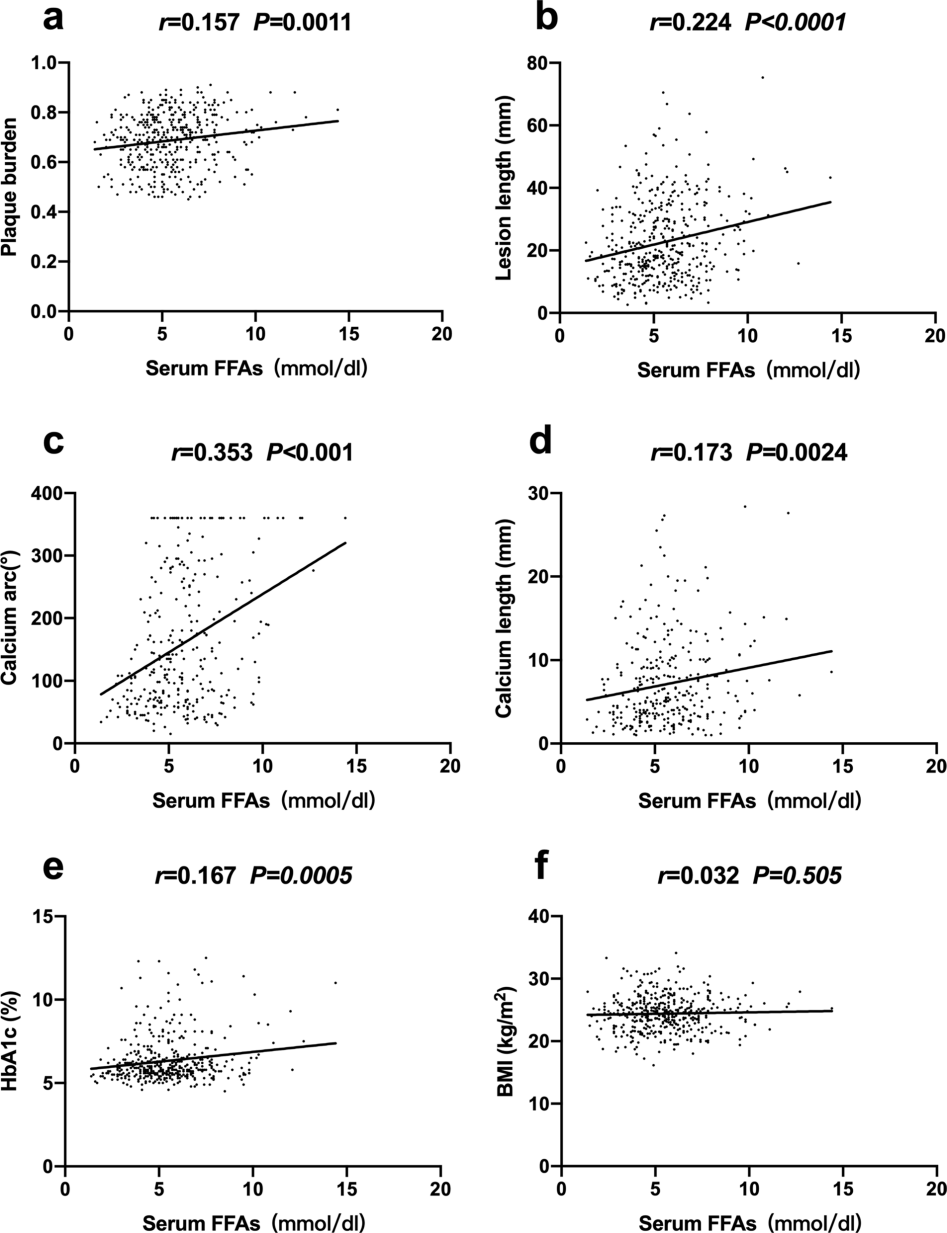
Pearson correlation analysis of serum FFAs with plaque characteristics (**a**, **b**), coronary calcification severity (**c**, **d**), HbA1c (**e**) and BMI (**f**)

Pearson’s correlation analysis revealed that serum FFA levels were significantly correlated with plaque burden (r = 0.157, *p* = 0.001), lesion length (r = 0.224, *p* < 0.0001), calcium length (r = 0.173, *p* = 0.002), calcium arc (r = 0.353, *p* < 0.001) and HbA1c (r = 0.167, *p* < 0.001). No positive relationship was found between serum FFAs and BMI (r = 0.032, *p* = 0.505) (Fig. 1).

### Correlation between serum lipid profiles and the extent of coronary calcification

**Fig. 2:**
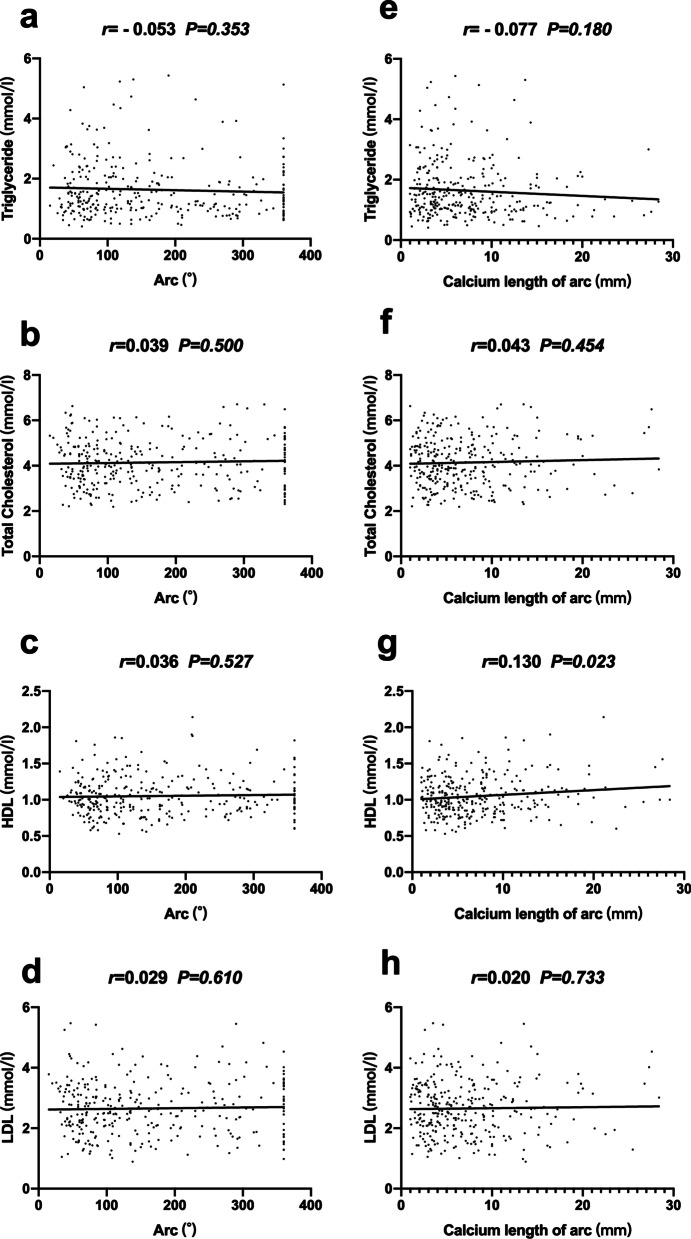
Pearson correlation analysis between calcified parameters and subfractions of serum lipid profiles. Calcified arc with lipids (**a**, **b**, **c**, **d**), calcium length of arc with lipids (**e**, **f**, **g**, **h**)

No positive correlations were found between subfractions of serum lipid profiles (triglyceride, total cholesterol, high density lipoprotein, low density lipoprotein) and calcium parameters (calcium arc, calcium length of arc), except for a weak correlation between high density lipoprotein and calcium length (r = 0.130, *p* = 0.023), (Fig. 2).

### Risk factors of SCAC in the whole cohort and subgroups

**Fig. 3:**
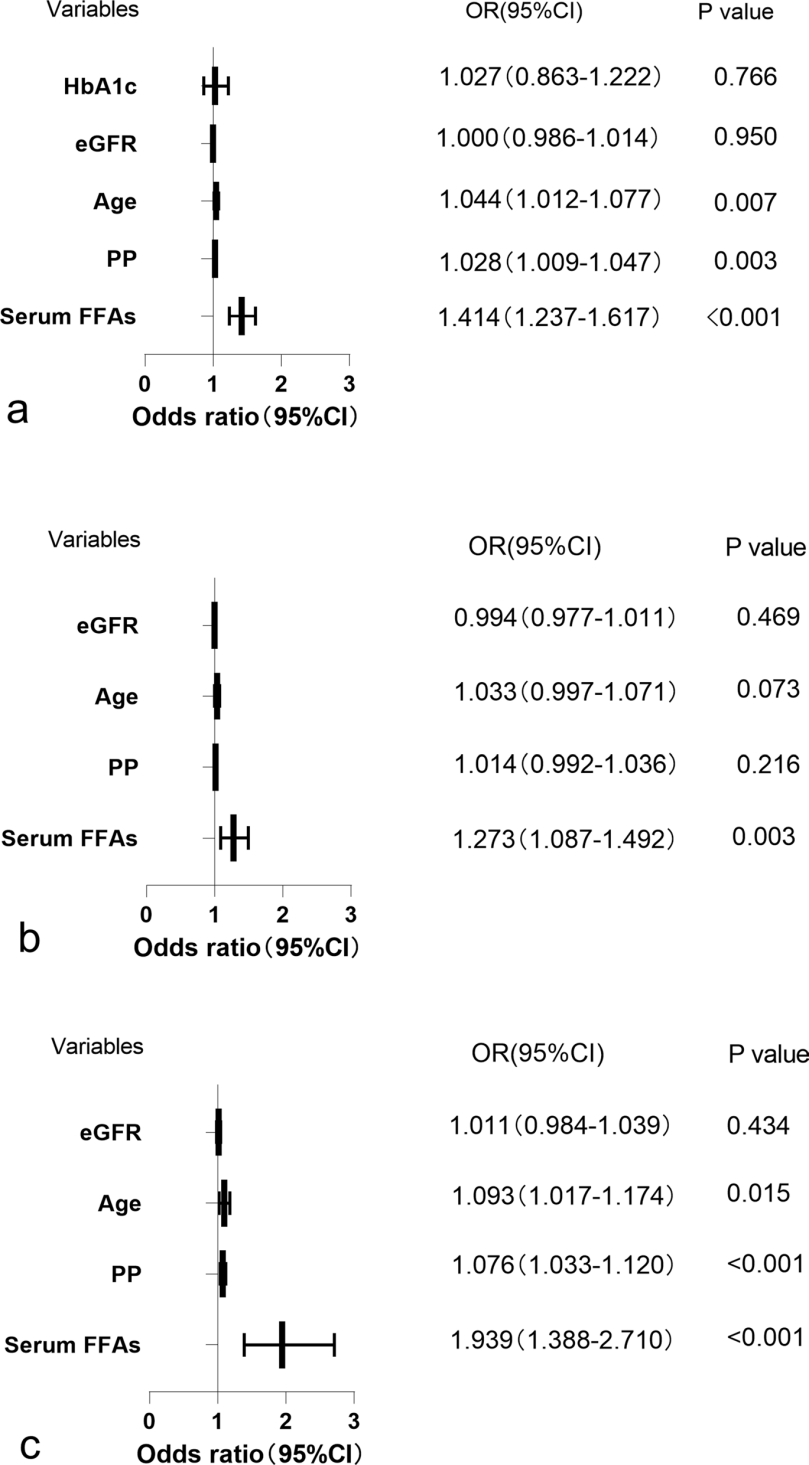
The Forest plot between SCAC and the risk factors in the whole population (**a**), non-DM subgroup (**b**) and DM subgroup (**c**). Values on the right side are reported as the odds ratio with the respective 95% confidence interval in parenthesis

All the variables were compared with independent t-tests or chi-square test. Potential confounders with a *p-*value < 0.10 combined with traditionally recognized risk factors of cardiovascular disease were entered into the logistic regression model. We built three models as presented in Table 3 and Fig. 3. Model 1 showed that in the whole cohort, after adjusting for pulse pressure, age, eGFR and HbA1c, serum FFA levels were independently associated with SCAC (OR = 1.414, 95% CI = 1.237–1.617, *p* < 0.001, Model 1), with a 41.4% increased odds of SCAC for every 1 mmol/dl increase in serum FFA. Model 2 revealed that in the non-DM group (n = 302), the relationship was weakened (OR = 1.273, 95% CI = 1.087–1.492, *p* = 0.003, Model 2). As for the DM group (n = 124), the association was the strongest (OR = 1.939, 95% CI = 1.388–2.710, *p* < 0.001, Model 3), with a 93.9% increased odds of SCAC for every 1 mmol/dl increase in serum FFA after adjusting for traditional cardiovascular risk factors. Serum FFA levels in the DM group were higher compared with the non-DM group (Additional file 1: Table S1).

**Table 3:** Multivariate logistic regression for SCAC in the whole cohort (Model 1), non-DM group (Model 2), DM group (Model 3)

	Model 1 (whole cohort)			Model 2 (non-DM)			Model 3 (DM)		
	OR	95%CI	Sig	OR	95%CI	Sig	OR	95%CI	Sig
Serum FFAs	1.414	1.237–1.617	< 0.001	1.273	1.087–1.492	0.003	1.939	1.388–2.710	< 0.001
PP	1.028	1.009–1.047	0.003	1.014	0.992–1.036	0.216	1.076	1.033–1.120	< 0.001
Age	1.044	1.012–1.077	0.007	1.033	0.997–1.071	0.073	1.093	1.017–1.174	0.015
eGFR	1.000	0.986–1.014	0.950	0.994	0.977–1.011	0.469	1.011	0.984–1.039	0.434
HbA1c	1.027	0.863–1.222	0.766						

PP, pulse pressure; eGFR, estimated glomerular filtration rate

### Predictive ability of FFAs for SCAC

**Fig. 4:**
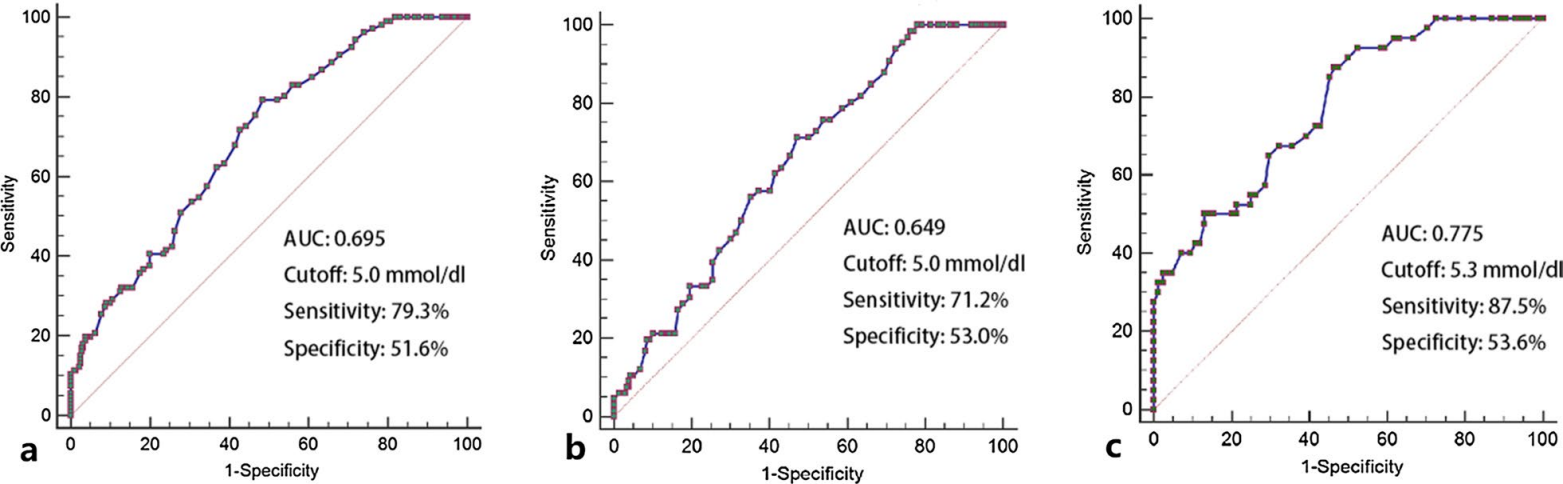
ROC curve analysis of Serum FFA levels in distinguishing SCAC in the whole population (**a**), non-DM subgroup (**b**) and DM subgroup (**c**)

The predictive ability of serum FFAs for SCAC was assessed by ROC curve analysis (Fig. 4). Similar to the logistic regression results, the AUC was 0.695 (CI 0.641–0.750, *p* < 0.001, cutoff value: 5.0 mmol/dl; sensitivity: 79.3%; specificity: 51.6%) in the total population. In the non-DM group, the AUC was 0.649 (CI 0.580–0.718, *p* < 0.001, cutoff value: 5.0 mmol/dl; sensitivity: 71.2%; specificity: 53.0%). The AUC value (0.775) was the highest in the DM group (CI 0.690–0.859, *p* < 0.001, cutoff value: 5.3 mmol/dl; sensitivity: 87.5%; specificity: 53.6%).

## Discussion

The main findings of the present study were as follows: (1) Serum FFAs were independently associated with SCAC, and the relationship was enhanced in the DM group. (2) DM patients had a higher risk of elevated FFAs and SCAC. (3) Serum FFA levels may have some predictive capacity for SCAC, especially in the DM subgroup.

The relationship between serum FFA levels and arterial calcification has been discussed in a few studies. Brooder MR et al. [22] found that saturated FFAs can increase medial calcification represented by arterial stiffness in a rat model. The results support our view, although they used palmitate, a component of FFAs abundantly present in serum. In contrast, Conway et al. [26] and Ormseth et al. [10] did not find any relationship between serum FFA levels and CAC. This discrepancy may be due to the inclusion criteria since Conway et al. [26] and Ormseth et al. [10] enrolled either younger T1DM patients or rheumatic patients, while we enrolled all patients with coronary artery disease in the real world. Furthermore, given that saturated FFAs enhance and unsaturated FFAs reduce vascular calcification and arterial stiffness [30, 31], we chose whole FFAs and evaluated the relationship between serum FFA levels and SCAC in our study.

Some previous studies examined the extent of CAC with CT [10, 26]. In this study, we used IVUS to examine the level of calcification. IVUS is the gold standard for CAC detection, with high sensitivity and specificity [9, 32]. Compared with CT, IVUS can also provide more details of calcification, such as morphology, position, arc and length [33], which increased the accuracy and reliability of the results of our study.

DM is correlated with CAC extent [2]. Our data showed that 37.7% of the patients in the SCAC group suffered from T2DM compared with 26.2% of the patients in the non-SCAC group. From our ROC analysis, serum FFAs showed certain predictive potential of SCAC, with the best in the DM subgroup. In line with this finding, Schauer et al. [11] reported that a high FFA level coupled with insulin resistance can predict the extent of CAC, and may contribute to the increased risk of cardiovascular disease in patients with T1DM. Experimental studies showed that a high level of serum FFAs was associated with insulin resistance and the development of DM [11–13]. Meanwhile, insulin resistance, an important characteristic of DM, plays a crucial role in the process of calcification in DM patients [11, 34]. Hence, we speculated that FFAs may promote the progression of CAC to an advanced stage by affecting the insulin activity in DM patients. This hypothesis needs further investigation.

In this study, in addition to FFAs, pulse pressure (PP) and age also served as predictors of SCAC, which was in line with previous studies [35, 36]. CKD, a traditionally recognized risk factor for CAC, was not found to be significantly related to SCAC in our study, which may be due to our exclusion of patients with stage 4 to 5 CKD or patients treated with dialysis who suffered greatly from SCAC [37, 38].

This study had some limitations. First, this was a retrospective, single-center study with a relatively small sample size, and we focused only on the most severe coronary vessel without evaluation of the other coronaries. Second, we collected only serum fasting FFAs without postprandial FFA levels or various components of serum FFAs.

## Conclusions

Our present study indicated that serum FFAs may have some potential for predicting the severity of CAC, especially in DM patients. More prospective studies with large sample size are needed to further demonstrate the predictive value of FFA in CAC.

## Supplementary Information

**Additional file 1: Table S1.** Baseline clinical characteristics between non-diabetic (Non-DM) and diabetic (DM) groups.

## Data Availability

The datasets analyzed in the study can be obtained from the corresponding author on reasonable request.

## Abbreviations

FFA: Free fatty acids
DM: Diabetes mellitus
CAC: Coronary artery calcification
IVUS: Intravascular ultrasound
SCAC: Severe coronary artery calcification
ROC: Receiver operating characteristic
AUC: Area under the curves
OR: Odds ratio
CI: Confidence interval
CKD: Chronic kidney disease
PCI: Percutaneous coronary intervention
CT: Computed tomography
CAG: Coronary angiography
OCT: Optical coherence tomography
CAD: Coronary artery disease
CABG: Coronary artery bypass grafting
NYHA: New York Heart Association
eGFR: Estimated glomerular filtration rate
BMI: Body mass index
HbA1c: Glycated hemoglobin
eGFR: Estimated glomerular filtration rate
CSA: Cross-sectional area
EEM: External elastic membrane
LA: Lumen area
MLA: Minimum lumen area
T1DM: Type 1 diabetes mellitus
