# Supplementary material for: Serum free fatty acids are associated with severe coronary artery calcification, especially in diabetes: a retrospective study
Source: BMC Cardiovasc Disord. 2021 Jul 15;21:343. doi: 10.1186/s12872-021-02152-w (PMC8281587; doi:10.1186/s12872-021-02152-w)
Supplement: Supplementary file 1 — Additional file 1: Table S1. Baseline clinical characteristics between non-diabetic (Non-DM) and diabetic (DM) groups. [file 12872_2021_2152_MOESM1_ESM.docx]

**Table S1.** Baseline clinical characteristics between non-diabetic (Non-DM) and diabetic (DM) groups.

| **Variable** | Non-DM  (n=302） | DM  (n=124) | ***P* value** |
| --- | --- | --- | --- |
| Age, yrs. | 66.54±10.72 | 68.60±9.01 | 0.061 |
| Male sex, % | 210 (69.5) | 71 (57.3) | 0.015 |
| BMI, kg/m^2^ | 24.33±2.87 | 24.59±2.79 | 0.400 |
| Smoking, % | 164(54.3) | 57(46.0) | 0.118 |
| Family history, % | 77(25.5) | 26(21.0) | 0.321 |
| Hypertension, % | 208(68.9) | 95(76.6) | 0.109 |
| Prior MI，% | 28 (9.3) | 14 (11.3) | 0.525 |
| Prior PCI % | 58(19.2) | 32(25.8) | 0.129 |
| Clinical presentation |  |  |  |
| SCAD | 253 (83.8) | 101 (81.5) | 0.561 |
| ACS | 49 (16.2) | 11 (18.5) |  |
| eGFR, ml/min/1.73m^2^ | 82.34±19.88 | 77.35±22.53 | 0.033 |
| HbA1c, % | 5.72±0.45 | 7.83±1.70 | 0.000 |
| TG, mmol/L | 1.58±0.86 | 1.76±0.96 | 0.063 |
| TC, mmol/L | 4.11±0.97 | 4.29±1.10 | 0.103 |
| HDL, mmol /L | 1.06±0.26 | 1.04±0.26 | 0.410 |
| LDL, mmol/L | 2.62±0.82 | 2.75±0.93 | 0.174 |
| Serum FFAs, mmol/dl | 5.28±1.85 | 6.07±2.11 | <0.001 |
| SBP, mmHg | 130.70±16.84 | 133.47±17.37 | 0.126 |
| DBP, mmHg | 75.46±10.36 | 73.94±10.89 | 0.176 |
| PP, mmHg | 55.23±13.36 | 59.61±13.83 | 0.002 |
| Prior medical treatment |  |  |  |
| Anti-platelet drugs (n/%) | 75 (24.9) | 44(35.5) | 0.027 |
| Statins (n/%) | 66 (21.9) | 33 (26.6) | 0.291 |
| ACEI/ARB (n/%) | 128 (42.4) | 66(53.2) | 0.041 |
| β-blockers (n/%) | 52 (17.2) | 29 (23.4) | 0.141 |
| CCB (n/%) | 92 (30.5) | 52 (41.9) | 0.023 |
| Insulin (n/%) | 0 (0.0) | 32 (25.8) | <0.001 |

Data are expressed as the mean ± SD. BMI, body mass index; MI, myocardial infarction; PCI, percutaneous coronary intervention; SCAD, stable coronary artery disease; ACS, acute coronary syndrome; eGFR, estimated glomerular filtration rate; TC, total cholesterol; TG, triglyceride; HDL, high-density lipoprotein cholesterol; LDL, low-density lipoprotein cholesterol; SBP, systolic blood pressure; DBP, diastolic blood pressure; PP, pulse pressure; ACEI/ARB, angiotensin-converting enzyme inhibitors/angiotensin receptor blockers; CCB, calcium channel blockers.
